# Collagen Functionalization of Polymeric Electrospun Scaffolds to Improve Integration into Full-Thickness Wounds

**DOI:** 10.3390/pharmaceutics15030880

**Published:** 2023-03-08

**Authors:** Aswathy Ravindran Girija, Xanthe Strudwick, Sivakumar Balasubramanian, Vivekanandan Palaninathan, Sakthikumar Dasappan Nair, Allison J. Cowin

**Affiliations:** 1 Future Industries Institute, University of South Australia, Mawson Lakes, SA 5095, Australia; 2 Bio Nano Electronics Research Center, Graduate School of Interdisciplinary New Science, Toyo University, Kawagoe 350-0815, Saitama, Japan

**Keywords:** poly(L-lactic acid), electrospun scaffolds, wound healing, collagen, biofunctionalization

## Abstract

Background: Electrospun fibers are widely studied in regenerative medicine for their ability to mimic the extracellular matrix (ECM) and provide mechanical support. In vitro studies indicated that cell adhesion and migration is superior on smooth poly(L-lactic acid) (PLLA) electrospun scaffolds and porous scaffolds once biofunctionalized with collagen. Methods: The in vivo performance of PLLA scaffolds with modified topology and collagen biofunctionalization in full-thickness mouse wounds was assessed by cellular infiltration, wound closure and re-epithelialization and ECM deposition. Results: Early indications suggested unmodified, smooth PLLA scaffolds perform poorly, with limited cellular infiltration and matrix deposition around the scaffold, the largest wound area, a significantly larger panniculus gape, and lowest re-epithelialization; however, by day 14, no significant differences were observed. Collagen biofunctionalization may improve healing, as collagen-functionalized smooth scaffolds were smallest overall, and collagen-functionalized porous scaffolds were smaller than non-functionalized porous scaffolds; the highest re-epithelialization was observed in wounds treated with collagen-functionalized scaffolds. Conclusion: Our results suggest that limited incorporation of smooth PLLA scaffolds into the healing wound occurs, and that altering surface topology, particularly by utilizing collagen biofunctionalization, may improve healing. The differing performance of the unmodified scaffolds in the in vitro versus in vivo studies demonstrates the importance of preclinical testing.

## 1. Introduction

Steadily turning into a major public health concern, the global skin and wound care market is expected to grow from USD 18.49 billion in 2017 to USD 25.98 billion by 2025 [1]. Wound dressings play a critical role in wound care management, primarily providing an environment that stimulates healing by preventing fluid loss and protecting against microbial contamination [2,3]. However, most current wound care dressings are not completely successful in promoting regeneration of skin. Therefore, various studies are presently aimed at the development of novel and efficient dressing materials that can accelerate the process of wound healing in various forms including pads, films, hydrogels, sponges, and micro and nanofibers. Amongst these forms of wound dressing, micro/nanofibers are remarkably attractive because they resemble the natural fibrous network structure of the extracellular matrix (ECM) [4,5] and function as a provisional support that facilitates the adherence and proliferation of cells to generate native ECM in the wound [6]. Several approaches have been established for the fabrication of biomaterial-based scaffolds as wound dressings, including solvent casting, freeze-drying, particulate leaching, rapid prototyping, and electrospinning [7,8]. The electrospinning technique in particular has the potential for developing scaffolds with tuneable diameter to resemble or mimic natural ECM, with a range of polymers commonly used in tissue engineering studies [9,10]. Utilising biodegradable and biocompatible polymers in combination with electrospinning enhances cellular interaction and growth due to their biocompatibility with wound tissue and blood. Parameters such as the scaffold architecture, mechanical characteristics, geometry, dimensions and surface topography and chemistry all can direct the behaviour of cells with respect to attachment, proliferation and differentiation to influence the complex cellular process involved in the formation of new tissue [11,12]. Thus, electrospun scaffolds have the potential to exhibit properties including an enhanced surface-area-to-volume ratio, better mechanical properties, improved porosity, and exceptional capability to deliver bioactive agents such as growth factors, antimicrobials, anti-inflammatory agents, drugs, or nanoparticles into the nanofibers, which in turn can promote skin regeneration and accelerate wound healing [13,14].

Collagen is the key component of the ECM of several tissues including the skin. During the process of wound healing, collagen not only provides mechanical support for tissue repair, but influences the synthesis of other ECM proteins and plays a vital role in regulating the inflammatory response via the release of inflammatory cytokines as well as growth factors that are involved in the remodelling of the ECM [15,16]. Cells interact with collagen and collagen stimulates the differentiation of cellular phenotypes during wound healing [17]. Owing to its beneficial properties, such as superior biocompatibility, reduced cytotoxicity and antigenicity, and ability to promote cell adhesion, proliferation and migration, collagen is widely incorporated into wound dressings [18]. Several collagen-based dressings in the form of films, gels, hydrogels, pads, powders, sheets, membranes, cellular matrices and nanofibers are available on the market and are also under research; these are based on pure collagen, blended with other polymers, or coated over other polymers [19,20]. Collagen modification or collagen-based electrospun scaffolds mimic the native ECM in the skin and stabilise cell and vascular components in the wound by decreasing the level of matrix metalloproteinases (MMP), which is imbalanced in deep or chronic wounds [15]. The porosity that arises during the process of electrospinning offers structural support for cells to develop new tissues in the wound [21]. Collagen-based electrospun scaffolds also have the ability to absorb large volumes of wound exudate and maintain moisture for the wound, thus promoting a superior wound-healing process [20,21,22,23].

While pure collagen-based scaffolds face the major limitations of a faster degradation rate and poor mechanical properties, blending collagen with other polymers (natural or synthetic) such as polyethylene oxide (PEO), PCL, poly (L-lactic acid) (PLLA), PVA, hyaluronic acid (HA), elastin (EL) and silk fibroin (SF) has significantly improved its mechanical properties and has been widely used in skin tissue engineering studies [24,25,26,27,28,29,30]. Collagen can be coated or grafted using different methods such as drop casting (adsorption) and cross linking, or by creating chemical bonding with activated carboxylic functional groups [31,32,33]. We have previously used a simple drop casting method to coat PLLA electrospun scaffolds with collagen, and have shown that the electrospun scaffolds with variation in surface topology and surface chemistry affected the cellular responses including adhesion, proliferation, differentiation, and migration of keratinocytes and fibroblasts, with collagen-modified scaffolds (both porous and smooth) producing confluent and uniform epidermal sheets of keratinocytes on one plane, with healthy fibroblasts populated within the scaffolds [34]. 

In this study, we investigated how our different electrospun PLLA fiber scaffolds integrate into wounds and act to support healing in vivo using a mouse full-thickness excisional wound model. We hypothesized that application of these scaffolds could dramatically improve the healing of deep skin wounds and assessed not only the integration and infiltration of cells into the scaffold, but also their effects on wound closure, re-epithelialization and extracellular matrix deposition.

## 2. Materials and Methods

### 2.1. Scaffold Preparation 

PLLA scaffolds were fabricated by an electrospinner (Nanon- O1A MECC Co. Ltd. Fukuda, Japan) with PLLA, (Mw of 80,000–100,000, Polyscience Inc., Warrington, PA, USA) [34]. Smooth fibers were fabricated by dissolving PLLA in 1,1,1,3,3,3-hexafluoro-2-propanol (HFIP) (Fujifilm Wako Pure Chemical Corporation, Osaka, Japan) to obtain a 14.5% *w*/*v* of PLLA solution using magnetic stirring for 4 h; this was followed by 4 h rest at room temperature (RT). The electrospinning process was performed at RT, a flow rate of 0.5 mL/h and an applied voltage of 15 kV. A grounded plate placed 12 cm from the tip of the needle was used as a collector. Porous fibers were fabricated using 11.5% PLLA solution with a binary solvent system of chloroform: dimethyl formamide (9:1) and stirred for 24 h. The solution was further allowed to rest for another 4 h before electrospinning. Electrospinning was performed at a potential difference of 15 kV with a flow rate of 2 mL/h. The grounded plate was placed 12 cm from the tip of the needle and was used as the collector. The air humidity and temperature conditions during the spinning process were about 40–50% and 25 °C, respectively. The mat from the collector was detached, dried to remove any residual solvents, and stored for further characterization studies.

Smooth- or porous-surfaced polymeric scaffolds were subsequently functionalized by a simple drop casting method with 0.1% collagen type I (Sigma Life Science, Darmstadt, Germany), as described in Aswathy et al., 2020 [31]. For collagen-modified scaffolds, both UV-sterilized smooth and porous PLLA scaffolds were soaked in 0.1% collagen for 30 min prior to application. Cocultures of keratinocytes and fibroblasts upon the scaffolds were prepared as previously described [34], and the morphology of the original polymeric scaffolds (before and after collagen coating) and the adhered cells were examined after 5 days’ coculture by scanning electron microscopy (SEM) using a Zeiss Merlin FEG SEM following previously described methods [34].

### 2.2. Excisional Wound Model

**Figure 1:**
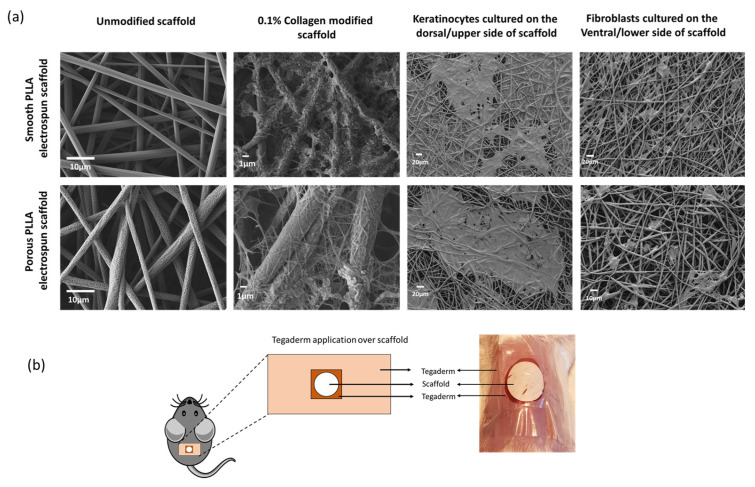
Collagen functionalization of polymeric electrospun scaffolds for wound healing applications. (**a**) SEM micrographs depicting the morphological features of unmodified smooth and porous, collagen-modified smooth and porous electrospun scaffolds and co-cultured skin cells on electrospun PLLA scaffolds on collagen-modified porous and smooth scaffolds. (**b**) Prepared 10 mm-diameter scaffolds were applied to the centre of full-thickness excisional mouse wounds and covered with the Tegaderm film as illustrated in the schematic and in the photograph.

The use of animals was approved by the University of South Australia’s Animal Ethics Committee (U15/20) following the Australian Code of Practice for the Care and the Use of Animals for Scientific Purposes. Power studies showed that a sample size of 6 (equal males vs. females for all polymeric electrospun scaffolds) would give 80% power using a 5% test level and a one-tailed test. Male and female BALB/c mice of 10 weeks old were procured from Animal Resources Centre, Perth, Australia. Following acclimatization for 5 days, mice were administered pre-operative analgesia (Buprenorphine 0.05 mg/kg) via subcutaneous (SC) injection 30 min prior to wounding. Anaesthesia was induced by isoflurane inhalation, and the dorsal side of mice was prepared by removing hair with an electric shaver followed by Veet depilatory cream; the area was then cleansed with sterile water and ethanol (70% *vol*/*vol*). Full-thickness excisional wounds were created on the midline (wounds at 1.5 cm from the base of the skull and 0.5 cm either side of the midline) using a 10 mm diameter sterile biopsy punch (Acu-Punch, Norwalk, CA, USA). UV-sterilized circular scaffolds of 10 mm were inserted into the wound to act as dermal scaffolds within the full-thickness excisional wound; these were held in place by Tegaderm dressings that had an additional square of Tegaderm the size of scaffold (10 mm) placed over the sticky side to prevent the Tegaderm sticking to the scaffold itself whilst providing covering and protection of the scaffold within the healing wound (Figure 1b). Post-operative analgesia (Buprenorphine 0.05 mg/kg) was administered subcutaneously just prior to the dark cycle on day 0 for pain relief. The mice were monitored daily, and the scaffold dressings were allowed to remain in place for the duration of the study, with the Tegaderm reapplied as required. The mice were humanely killed by CO_2_ inhalation at 7 or 14 days post wounding, and the wounds were assessed for healing as described below. 

### 2.3. Macroscopic Wound Assessment

Digital photographs were taken on day 0, 7 and 14 with a ruler aligned next to the wound. Image Pro Plus image analysis software (Media Cybernetics, Inc., Bethesda, MD, America) was used to measure the area and diameter across the midline of the wounds, which were calibrated against the 1 mm graduations of the ruler that was included in the frame of each image. Healing at day 7 and 14 calculated by normalization to the day 0 wound using the following formula:

$$\frac{\mathrm{Measurement}\;\mathrm{of}\;\mathrm{initial}\;\mathrm{wound}-\mathrm{measurement}\;\mathrm{of}\;\mathrm{actual}\;\mathrm{wound}}{\mathrm{Measurement}\;\mathrm{of}\;\mathrm{initial}\;\mathrm{wound}}\times 100\%$$

High resolution images of the healed wound on day 14 were also obtained using the DermaScope unit of the DermaLab Combo (Cortex Technology) following the manufacturer’s instructions. Erythema within the wound area was also assessed using skin reflectance spectroscopy by the DermaLab Combo to determine the redness of vascularized or inflamed skin. To obtain the erythema measurement, the instrument was calibrated and the reading taken directly above the wound to obtain an open-ended index in CIELab colour values and normalized to an area of unwounded dorsal skin for each mouse. Transepidermal water loss (TEWL) as an indicator of restored skin barrier function was measured for 8 s per site using the Vapometer evaporimeter (Delfin Technologies, Kuopio, Finland), following the manufacturer’s guidelines. Using an 11 mm adaptor, the Vapometer was placed directly onto the wounded or adjacent unwounded dorsal skin, and in the wound to obtain the TEWL in g/m^2^/h.

### 2.4. Histological Assessment of Healing

The entire wound at day 7 or 14, including the scaffold and adjacent normal skin, was excised from the back of the mice and fixed in a formaldehyde-buffered solution (10% Neutral Buffered Formalin, Sigma) and embedded in paraffin wax for histological analysis. Histological sections of 4 μm were stained with haematoxylin and eosin (H&E) or Masson trichrome using previously described methods [35], and images were acquired using an Olympus IX81 light microscope (Olympus, Tokyo, Japan). The wound width and panniculus gape were manually quantified using Image Pro Plus image analysis software. The percentage re-epithelialization (%) was calculated based on the total wound length (the area between the first hair follicle either side of the wound and above the break in the panniculus) at day 7, using the following formula:

 Re−epithelialized wound lengthTotal epithelialized+un−epithelialized wound length×100%

Infiltration of cells into the scaffold within the wound was represented as a score from 0 to 5 (none to high) based on H&E staining and visualization of blue/purple nuclei within the scaffold. The images were then analyzed using cellSENS Microscope Imaging Software (Olympus) to quantify the number of cells per area by counting the nuclei within the scaffold. Matrix deposition was also given a score from 0 to 5 by monitoring the density of the extracellular matrix within the wound in each test group from H&E images. For quantitative morphometric analysis of collagen deposition, RGB images of Masson Trichrome-stained section were analyzed in Image J software (Version 1.32j, National Institutes of Health, Bethesda, MD, USA) using a macro written by Kennedy et al., 2006 [36], whereby the number of blue/green pixels indicating collagen with substantially greater (>120%) blue than red intensity are attributed the new, grey scale amplitude = 1, leaving other pixels = with amplitude = 0. Both the centre of the wound (defined ROI of standardized size) and the total wound area were measured. 

### 2.5. Statistical Analysis

All data are displayed as mean ± standard error of mean (SEM). The one-way analysis of variance test was used to assess the statistical comparisons. A value of *p* < 0.05 is considered statistically significant. All statistical analysis was performed using GraphPad Prism version 8.0 (GraphPad, Sacramento, CA, USA)

## 3. Results and Discussion

The morphology of smooth and porous PLLA electrospun fibers before and after collagen modification and subsequent coculture with keratinocytes and fibroblasts was assessed by SEM (Figure 1a). Unmodified smooth PLLA fibers exhibited random distribution (size ranging from 900–1200 nm). Unmodified porous fibers were in the range of 1000–1300 nm, with an average pore size of 50 nm. Collagen modification further altered the morphology and porosity of both smooth and porous fibers, and deposits of collagen can be seen as aggregates and ultrafine fibrous structures. When fibroblast cells were cultured on collagen-modified fibers, the cells exhibited spindle-like elongated patterns, whereas keratinocytes exhibited a uniform patch of cells with good cell–cell interactions. As discussed in our in vitro study [34], while the unmodified smooth scaffold displayed better cell adhesion than unmodified porous scaffolds, collagen-modified scaffolds (both porous and smooth) produced confluent and uniform epidermal sheets of keratinocytes on one plane, with healthy fibroblasts within the scaffolds. Collagen-modified electrospun smooth and porous PLLA scaffolds of 10 mm diameter and unmodified counterparts were applied to the mice on the day of injury; full-thickness excisional wounds beneath a Tegaderm dressing (Figure 1b) were used to assess their ability to improve wound healing. 

**Figure 2:**
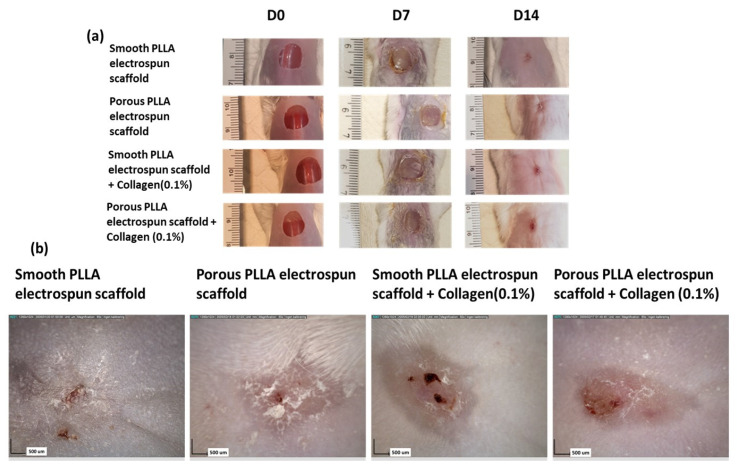
Macroscopic wound assessment following treatment with unmodified or collagen-functionalized smooth and porous PLLA scaffolds. (**a**) Representative images of wounds from the study groups: smooth PLLA electrospun scaffold, porous PLLA electrospun scaffold, smooth PLLA electrospun scaffold + collagen (0.1%) and porous PLLA electrospun scaffold + collagen (0.1%) at day 0, 7, and 14 (all images include a ruler with small graduations of 1 mm). (**b**) Wounds were also imaged under Dermascope at day 14 (scale bar 500 µm).

Wounds were imaged on the day of surgery prior to scaffold application (day 0) and again on days 7 and 14 post-surgery (Figure 2a). No signs of any infection or major inflammation in the wounds were observed when the electrospun PLLA scaffolds were applied to the wounds. The scaffolds remained visibly attached to the wound at day 7 but were not observed by day 14. The wounds in all the treatment groups (smooth PLLA, porous PLLA, collagen-modified smooth PLLA and collagen-modified porous PLLA electrospun scaffolds) were not well healed on day 7, while the wounds were completely closed on day 14. Representative photographs from all treatment groups are provided in Figure 2. Images from Dermascope provided high-resolution images of healed wounds (Figure 2b). Wounds were healed in all the treatment groups. The PLLA scaffolds treatment groups did not exhibit any scabs, and exhibited healing with minimal scarring evident.

**Figure 3:**
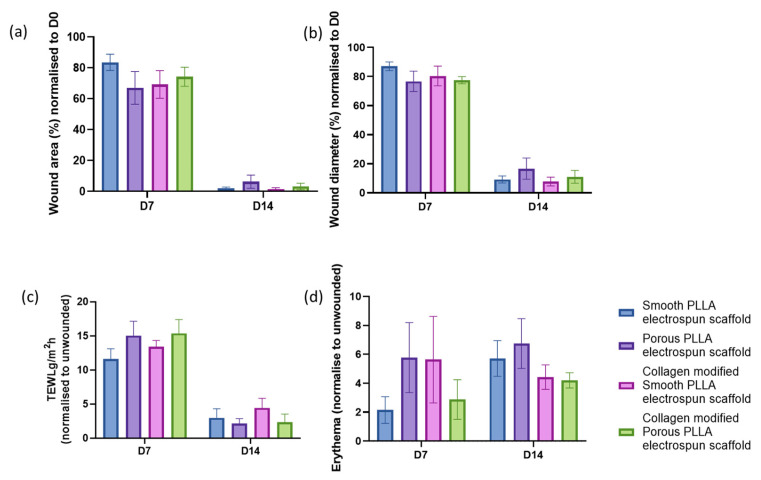
Quantification of macroscopic healing. Wound area (**a**) and diameter across the midline (**b**) of excisional wounds at days 7 and 14, normalised to day 0 and expressed as a percentage of initial (%). (**c**) Transepithelial water loss (TEWL) through the wound on day 0 and 7. Data expressed as g/m^2^/h normalized to unwounded skin. (**d**) Wound redness (erythema) measured on day 7 and day 14 and normalized to adjacent unwounded skin. Bars represent mean ± SEM, n = 6 mice.

Quantification of the macroscopic wound area (Figure 3a) and diameter (Figure 3b) suggested at day 7 that smooth, unmodified scaffolds were the worst performing and unmodified porous scaffolds were the best; however, by day 14, the mice treated with unmodified porous scaffolds had the largest wound area and diameter, with no significant differences between any of the groups found. TEWL measurements were taken to evaluate barrier function during the treatment of excisional wounds with an electrospun scaffold as a dressing (Figure 3c). No significant differences in TEWL were observed, although at day 7, both the unmodified and collagen-modified porous scaffold treatment groups had slightly higher TEWL than the two smooth scaffold groups, with the unmodified smooth scaffold-treated wounds having the lowest water loss. By day 14, however, when all groups showed low TEWL—which is indicative of the newly reinstated skin barrier—the opposite trend was observed, with the two porous scaffolds now having the lowest TEWL compared to the two smooth scaffold groups. No significant differences in wound redness were found, with wounds treated with smooth, unmodified scaffolds having the lowest erythema at day 7, and the unmodified porous and collagen-modified smooth scaffolds having the highest; however, by day 14, the unmodified porous group had the highest and the two collagen-modified scaffold groups exhibited the lowest redness within the wound. In contrast to wound area, diameter and TEWL—which all reduced over time as healing progressed—the erythema scores increased overtime, indicating that the measure is representative of vascularization within the healing wound tissue increasing as the wounds healed.

**Figure 4:**
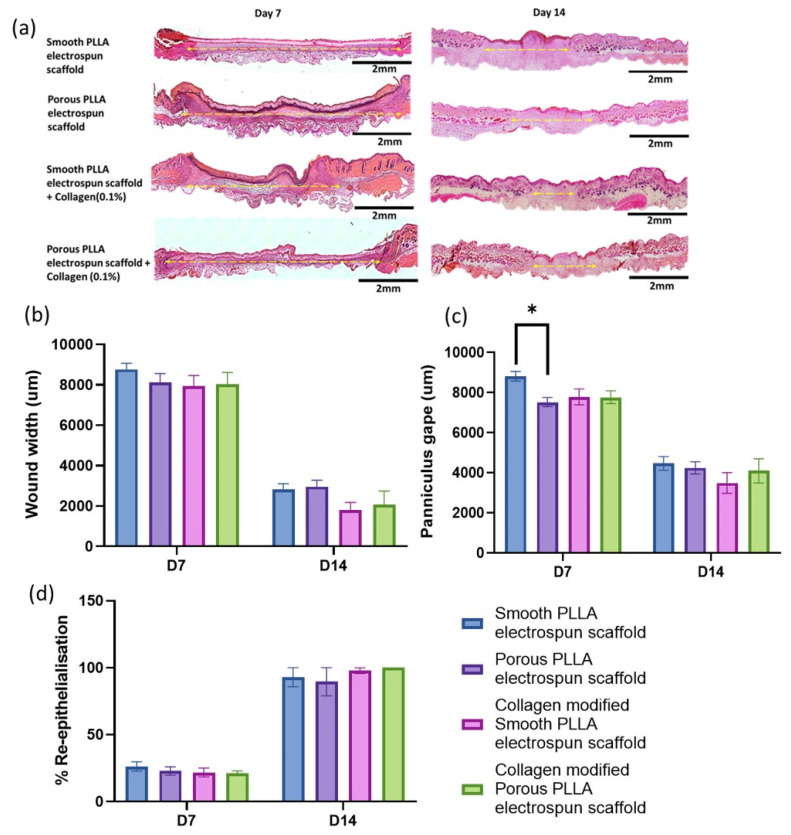
(**a**) Representative images of smooth PLLA electrospun scaffold, porous PLLA electrospun scaffold, smooth PLLA electrospun scaffold + collagen (0.1%) and porous PLLA electrospun scaffold + collagen (0.1%) wound sections after H&E staining at days 7 and 14 of healing. Images captured at 10 × objective. Scale bar = 2 mm. Yellow dashed lines indicate wound width measured. The histological analysis of wounds demonstrates decreased microscopic wound width (**b**) and decreased panniculus gape (**c**) and increased average re-epithelialisation (**d**) in scaffold treated wounds. All data are represented as mean ± SEM, n = 6 mice, * = 0.03.

The histological evaluation of wound healing was subsequently performed on H&E-stained tissue sections from wounds of mice treated with all four PLLA-based scaffolds at day 7 and 14 (Figure 4a). Wound healing was evaluated using the Image Pro Plus software to determine the wound width (Figure 4b), panniculus gape (Figure 4c) and the percentage of re-epithelization (Figure 4d) at the centre of the wound. It was observed that at day 7, the new epidermal layer had not been formed yet in any group, and the wound was covered with electrospun scaffold dressing; however, the scaffold was no longer detectable in day 14 wounds. In previous studies, with our collagen-coated PLLA fibers, which had an average of 80 um (measured with a Nikon Digi micro MH-15M that has a 0–15 mm measuring range and a 0.01µm minimum readable value at an accuracy of 0.7 µm), we observed that in vitro degradation showed a weight loss of 17% and 23% in PBS at 37 deg for 15 and 30 days, respectively [37]. For this in vivo study, knowing that reepithelialisation of 10 mm excisions should be completed at around 14 days, we reduced the electrospinning time from 30 min to 10 min to reduce the thickness of fibers, now at around ~10–15 um. Furthermore, in vivo polymer degradation occurs much faster than in in vitro conditions due to oxidative degradation [38,39,40,41]. Therefore, considering our previous experience in vitro and our current results, the reduced thickness and the oxidative stress around the wound site may have led to the accelerated degradation of the scaffold, the manner of which may not be possible under ambient conditions and lower humidity. The absence of a neo-epidermis at day 7 indicates that the TEWL results at this timepoint are indicative of the scaffold’s ability to provide barrier function, with the day 14 results better indicating the ability of the scaffold to induce better restoration of the skin barrier. Once again, the early indications from all three histological parameters on day 7 suggested poorest healing following treatment with the unmodified smooth scaffold (with a significantly larger panniculus gape in this group compared to the unmodified porous scaffold on day 7). By day 14, however, healing was similar in this group to that of both the unmodified porous and the collagen-modified porous scaffolds, both of which had the highest epithelialization, suggesting that these wounds were more fully healed overall. 

**Figure 5:**
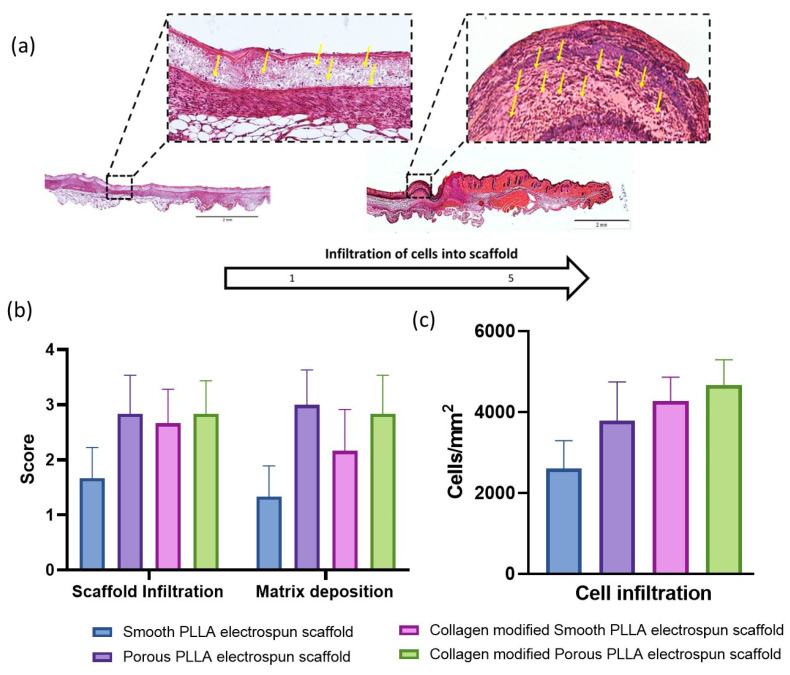
Infiltration of cells into and matrix deposition around the scaffold represented as a score from 0–5. (**a**) 100 X magnified images of representative scaffolds, with low to high infiltration and deposition. Scale bar = 2 mm. Yellow arrow indicating cellular infiltration. (**b**) A score of 1 was given to the scaffold with lower infiltration, as viewed by fewer nuclei or a pink-stained matrix, as opposed to more nuclei or matrices, which were given a score of 5. (**c**) Cell infiltration is represented as nuclei count (cells/mm^2^) in scaffolds. All data are represented as mean ± SEM, n = 6 mice.

The trends observed suggest that collagen coating impacts healing potential regardless of the surface topography of the original scaffold, which agrees with previous observations that synthetic scaffolds have limited cellular infiltration in their native form without modification [42]. Furthermore, several studies have established that the structural and functional regeneration of the skin upon any structural damage, such as large and deep wounds, depends largely on the regulation of the ECM deposition [43]. Indeed, it is the formation and deposition of collagen that is a primary signature of the later phases of wound healing, and is critical for the normal functioning of skin [44]. In our own previous in vitro study, we demonstrated that the addition of collagen to a porous scaffold resulted in the formation of a nanofibrillar structure [31], which might have further aided the cells to interact, proliferate and deposit ECM to heal the wounds. These in vitro studies showed good infiltration of fibroblasts into all scaffolds, and as such, we anticipated similar observation when scaffolds were used as dressings for excisional wounds in mice. Figure 5a depicts the representative image of the lowest score (1), demonstrating limited infiltration of cells and no matrix deposition, and the highest score (5), showing maximum cellular infiltration and ECM deposition. It was observed that not all the treatment groups exhibited infiltration of cells and supported deposition of ECM around the scaffold (Figure 5b), with histological assessments for scoring the cellular infiltration into the scaffold and deposition of extracellular matrix within the wound showing that both the unmodified porous scaffold and collagen-modified porous scaffold exhibited superior cellular infiltration and matrix deposition. Quantification of cell infiltration by nuclei count (Figure 5c) confirmed that the smallest number of cells were present within the smooth scaffolds, and that the collagen-modified scaffold had the highest density of cells. This may be co-connected to the topography of the scaffolds, owing to the roughness imparted by the porous scaffold itself and also to the nano-structured fibrillar structure arising from the collagen modification of the scaffold prior to application to the wound. The Masson trichrome collagen deposition results, wherein porous PLLA scaffold exhibited superior collagen deposition at day 7 and day 14 treatment, also support this observation.

**Figure 6:**
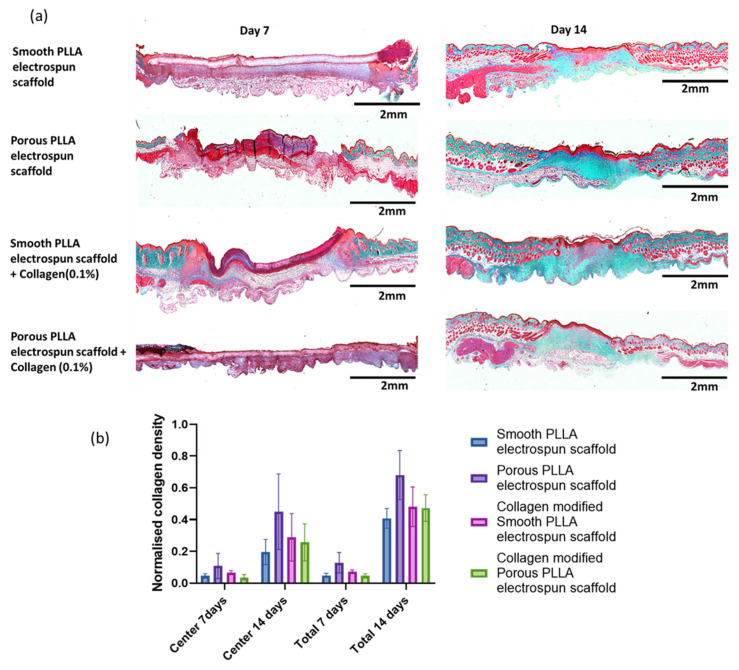
Collagen deposition analyzed by Masson’s trichrome staining. (**a**) Representative pictures of Masson’s trichrome staining showing collagen deposits (blue-stained) on day 7 and day 14, with various electrospun scaffolds as dressings. Scale bar = 2 mm. (**b**) Collagen deposition (normalized to unwounded skin) in the centre of the wound, and total wound on day 7 and day 14 on various scaffold treatments. All data are represented as mean ± SEM, n = 6 mice.

Masson’s Trichome staining, wherein collagen fibers, the primary ECM component, [44] are stained in blue/green colour; this was carried out to evaluate the collagen formation and distribution in the healing wound on day 7 and 14 (Figure 6a), where the relative intensity of blue staining corresponds to the amount of collagen deposited and indicates the progression of collagen synthesis and remodeling within the wound tissue (Figure 6b). Our results suggest that among the treatment groups, the porous PLLA scaffold had the most improved collagen synthesis both in center of the wound and throughout the total wound area on day 7 and day 14, and the rate of collagen fibers’ synthesis and deposition throughout the wound was lowest with thesmooth PLLA scaffold on both day 7 and 14. We anticipated collagen coating over the scaffold might trigger further collagen deposition; however, while both collagen-modified scaffold types had a slightly higher collagen deposition when compared to the smooth scaffold, the unmodified porous PLLA scaffold supported the greatest collagen deposition in the wounds.

## 4. Conclusions

In this study, electrospun PLLA scaffolds of different topologies (porous and smooth) were fabricated and functionalized with one of the major ECM proteins, collagen type I, and employed as wound dressings in mouse excisional wounds. Our previous in vitro studies showed that collagen modification enhanced major cellular activities including cell adhesion, spreading, proliferation and migration when skin cells were grown on the scaffolds, suggesting that collagen-modified electrospun scaffolds had the potential be used as promising wound-dressing materials to support a variety of cells and accelerate wound healing. Early indications confirmed that unmodified, smooth PLLA scaffolds may perform the worst in vivo, with these wounds having consistent trends towards delayed healing, appearing to be the largest in area, having the greatest wound width (including a significantly larger panniculus gape), and the lowest re-epithelialization. By day 14 however, no significant differences in healing were observed between the scaffolds. Nevertheless, wounds treated with the unmodified, smooth PLLA scaffolds still appear to have the least functional healing, with the lowest collagen deposition within the wound site. Taken together, the trends we have observed suggest that the limited incorporation of smooth PLLA scaffolds into the healing wound may be overcome by altering surface topology or biofunctionalization, and that in general, collagen biofunctionalization of PLLA scaffolds with either smooth or porous topology offers the greatest improvement to healing, with the collagen-functionalized smooth surfaces having a slightly decreased wound area and width compared to all other scaffolds, and collagen-functionalized porous scaffolds showing a decreased wound area and width when compared to the non-functionalized porous scaffold. Similarly, re-epithelialization at 14 days post-wounding was also highest in wounds treated with collagen-functionalized scaffolds. This trend towards faster healing in wounds in which scaffolds are modified prior to application may present some clinical advantages, such as protection from infection, due to a faster re-establishment of barrier function and reduction in the overall area; however, as no significant differences in the healing outcome of these uninfected wounds were observed, alternate avenues for improving the ability of electrospun scaffolds to enhance wound healing are required. Moreover, the opposing performance of the unmodified scaffolds in the in vitro versus this in vivo study demonstrates the importance of preclinical testing where unexpected results can occur.

## Data Availability

Data are contained within the article.
